# Prophylactic use of antibiotic-loaded bone cement in primary total knee arthroplasty: Justified or not?

**DOI:** 10.4103/0019-5413.53456

**Published:** 2009

**Authors:** Amit K Srivastav, Biren Nadkarni, Shekhar Srivastav, Vivek Mittal, Shekhar Agarwal

**Affiliations:** Delhi Institute of Trauma and Orthopaedics, Sant Parmanand Hospital, New Delhi, India

**Keywords:** Antibiotic-loaded bone cement, prophylaxis, total knee arthroplasty

## Abstract

**Background:**

The routine use of antibiotic-loaded bone cement (ABLC) during primary or uninfected revision arthroplasty remains controversial. Many studies quote the total joint arthroplasty (TJA) infection rate to be less than 1%. Total knee arthroplasty (TKA) has a higher infection rate than total hip arthroplasty (THA). Based on both animal and human studies in the past, ABLC has been found effective in reducing the risk of infection in primary TJA. We are presenting retrospective analysis of results in terms of infection rate in 659 TKA performed by a single surgeon under similar conditions during 2004–2007 using CMW1 (Depuy, Leeds, UK) with premixed 1 g of gentamicin.

**Patients and Methods:**

We did primary TKA in 659 knees of 379 patients during 2004–2007 using CMW1 (Depuy, Leeds, UK) cement containing 1 g of gentamicin in 40 g of cement in a premixed form. Standard OT conditions were maintained using laminar air flow, isolation suits for the operating team, pulse lavage and disposable drapes in each patients. Midvastus approach was used in all the patients to expose the knee joint. A systemic antibiotic (third-generation cephalosporin and aminoglycoside) was used preoperatively and 48 h postoperatively. We observed the patients in terms of infection in the high-risk and low-risk group till the recent follow-up with a mean of 20.6 months (9–38 months).

**Results:**

We had deep infection in six knees in six patients and all of them required two-stage revision surgery later in the high-risk group. Infection occurred at a mean of 20.5 months after surgery earliest at 9 months and latest at 36 months after surgery. The infection rate in our study was 0.91% which is comparatively less than the reported incidence of 1–2% in reported studies.

**Conclusion:**

We conclude that the use of antibiotic loaded bone cement is one of the effective means in preventing infection in primary TJA.

## INTRODUCTION

Much has been written about the use of antibiotic-loaded bone cement (ABLC) in the treatment of infected joint arthroplasty, but its use for prophylaxis against bacterial infection is less prevalent. A literature review of 1299 primary exchange revisions revealed that 99% of infected total hip arthroplasty surgeries were performed with antibiotic bone cement.^1^ Heck *et al*.^2^ surveyed 1015 surgeon responses from across the United States; out of them 56% used antibiotic cement in their practices. Of those, however, >90% used antibiotic-impregnated cement as prophylaxis in primary arthroplasty of previously infected joints. For routine primary total joint replacement, however, 11% of respondents regularly used antibiotic bone cement.

The Scandinavian Joint Registries report the prophylactic use of antibiotic cement in 95% of revision hip or knee arthroplasty,^3^,^4^ and a variation in primary joint replacement (48% in Norway,^4^,^5^ 85% in Sweden^3^). Fish *et al*.^6^ surveyed 338 American hospital pharmacists in 1992. They found that ABLC was used in urban settings, particularly when hospitals were affiliated with training programs.

We did retrospective study to know whether ABLC in primary total knee arthroplasty (TKA) is effective in reducing the rate of infection or not by using CMW1 (Depuy, Leeds, UK) with premixed 1 g of gentamicin per 40 g of cement in all the patients as routine along with a systemic antibiotic. We are presenting retrospective analysis of results in terms of infection rate in 659 TKA performed by single surgeon under similar conditions during 2004-2007 using CMW1 (Depuy) with premixed 1 g of gentamicin.

## PATIENTS AND METHODS

This is a retrospective study on patients operated between 2004 and 2007. A total of 659 knees were operated and primary TKA was performed by a single surgeon in 379 patients with degenerative osteoarthritis in 547 knees and 112 rheumatoid knees using similar technique in all the patients. Patients with diabetes, older than 75 years, immunocompromised patients including those taking steroids and those with immune disorders like rheumatoid arthritis, obese patients with BMI<30, patients having a history of a previous major knee surgery and those with a history of prior infection of the knee joint had been considered as the high-risk group. Average age of the patient was 63.3 years (46–88 years) including 198 males and 181 females. The mean body mass index (BMI) was 23.6 (17.5–37). A total of 120 knees in 70 patients had controlled diabetes mellitus, 80 knees in 40 patients had rheumatoid polyarthritis, 32 knees in 16 patients had both diabetes and rheumatoid arthritis. A total of 68 knees including 20 patients in the rheumatoid group with a bilateral involvement and another 28 knees in 14 patients with a bilateral involvement had a history of steroid intake for skin and other immune disorders. A total of 30 knees in 20 patients were obese having BMI > 30. Six knees had previous tubercular infection of the knee joint with a mean duration of 24.6 months (13 months–6 years) of completion of antitubercular treatment before presenting to us and all were healed when taken up for surgery. Ten knees in eight patients had a history of previous knee surgery; out of them six were arthroscopic and four were major open surgeries [Table 1]. Thus, two groups were made including 318 knees in 166 patients (120 DM + 80 RA + 28 steroid + 32 DM and RA + 6 previous tubercular infection + 4 prior major surgeries + 30 obese + 18 knees > 75 years of age) with a high risk and 341 knees in 183 patients without any risk factor [Table 1].

**Table 1:** Number of knees in the high and low-risk group

	High risk	Low risk
DM	120	
RA	80	
DM + RA	32	
Steroid intake (other than the rheumatoid group)	28	
Obese	30	
Previous infection	6	
Previous major surgery of the knee joint	4	
Age > 75	18	
Total	318	341

The mean preoperative clinical score was 43.4 (40–57) as per the HSS knee score.

After medical evaluation, 315 patients were given combined epidural and spinal anaesthesia, and general anesthesia was given to 64 patients. The duration of surgery ranged from 45 to 70 min in unilateral cases (mean 57.5) and 130 to 150 min (mean 140) in bilateral cases. Tourniquet was used in all the patients. Ideal OT conditions were maintained in all patients. Laminar air flow, isolation suits for surgeon, a preoperative and postoperative systemic antibiotic (third-generation cephalosporin and aminoglycosides) for 48 h were given to all the patients. Standard midvastus approach was used in each patient. We used gentamicin-loaded CMW1 (Depuy) cement containing 1 g of premixed gentamicin per 40 g of bone cement in all the patients after making sure that none of them were having allergic reaction of any kind in the past. One pack of cement was used in all the patients and was mixed manually. The components were cemented at the same instance in each of the patients. Pulse lavage was done in each surgery and wound was closed over a suction drain without applying any negative pressure. The amount of blood loss in suction drains was 350 ml (range 300–400 ml).Wound hematoma developed in 12 patients but resolved without any complication. Staples were removed on the 14^th^ postoperative day. Supervised physiotherapy for first 3 weeks including continuous passive motion (CPM) in initial days was followed by unsupervised range of motion and quadriceps' strengthening exercises. Mechanical prophylaxis was used mainly to prevent deep vein thrombosis (DVT), and low-molecular-weight heparin was given in 48 patients where a previous history of DVT was positive and in patients who were not cooperative enough for mechanical measures. Patients were followed up for a mean period of 20.6 months (9–38). Patients were followed up at 2 weeks, 6 weeks, 3 months, 6 months, and at 1-year interval afterward. Postoperative functional score improved to a mean of 93.4 (79–100) as per the HSS score. Diagnosis of infection was made by clinical (pain out of proportion, fever, local swelling, restricted range of motion, and aspiration of pus from the joint), hematological (increased total leukocytes' count with polymorph preponderance, increased ESR, increased CRP), increased uptake on bone scan and radiological evidence of component loosening. Acute infection included local complications as delayed wound healing, skin slough, or hematoma formation within 3 weeks of surgery. Intermediate or delayed infections were those resulting from contamination that occurred during the perioperative period or from hematogenous spread. Late deep infections are hematogenous spread of microorganism from distant sources. For the evaluation of the result, all the patients were included till recent follow-up ranging from 9 to 38 months.

## RESULTS

We had overall delayed deep infection in six (0.91%) knees in six patients including three females and three males from the high-risk group. Infection occurred at a mean of 20.5 months after surgery earliest at 9 months and latest at 36 months after surgery [Table 2]. None of the patients had infection those were without any risk. Three (2.5%) of them were diabetic and 3 (3.75%) of them were having rheumatoid arthritis. One patient with underlying diabetes mellitus which had developed infection was treated for an intercondylar fracture of the femur in the past and was having no signs of infection either in the past or at the time of TKA. The odd ratio was infinite (∞) as we had no infection in the low-risk group which shows that risk factors are statistically significant for causing infection.

**Table 2:** Number of knees that got infected in each group

	Total	Infection	Noninfection
High-risk	318	6	312
Low-risk	341	None	341

Open lavage failed in all the six knees and no organism could be grown. All of them required two-stage revision surgeries subsequently and vancomycin was given to all the six patients systemically as well as in bone cement. Till the recent minimum follow-up of 26 months following revision surgery, none of them is having any sign of recurrence of infection.

## DISCUSSION

Deep infection is a debilitating complication of TKA and has been cited as the most common cause of failure of an implant.^6^–^10^ Many contributory factors have been implicated such as patient factors (local skin condition, systemic medical condition, and prior surgery, etc.), operative factors (operating environment, operation course, operation suits, etc.), surgical technique (implant selection, antibiotic usage, postoperative wound care, etc.), and postoperative factors.^7^–^11^ Patients with diabetes, patients older than age 75 years, immunocompromised patients including those taking steroids and those with immune disorders, obese patients, and patients with hemophilia have higher infection rates. As the population ages, the indication for TJA expands to include these higher risk groups, and increased rates of infection are inevitable. Patients and society expect excellent outcomes in TJA, and surgeons desire to minimize the risk of infection, which has led to the use of ABLC in primary TJA.

In 1969, Buchholz *et al*.^12^–^14^ introduced the technique of combining antibiotics with bone cement. Palacos bone cement containing gentamicin powder was introduced as a commercial product in 1970. CMW bone cement containing gentamicin was introduced in 1990. ABLC serves as a form of prophylaxis for the prevention of infection in TKA.^15^ A conclusion made by Jiranek *et al*.^16^ supports the fact that Staphylococcal species are the primary bacteria toward which ABLC would be directed. The currently available commercial gentamicin bone cement provides sufficient elution concentration to be bactericidal even against methicillin-resistant organism. In addition, gentamicin is available in a premixed form, has low-allergy profile and the level of gentamicin in the joint is often 10 times greater than safe blood levels, and the efficacy is excellent.

The Swedish Knee Registry reports an infection rate of 1.7% in patients with osteoarthritis and 4.4% in patients with rheumatoid arthritis.^17^ ABLC is effective in reducing the risk of infection in primary TJA, based on both animal and human studies.^18^ A study comparing infection rates among 340 patients with and without cefuroxime in bone cement found statistically fewer infections with ABLC (*P* < 0.02)^18^. Animal studies using canine and rabbit models showed that when compared to cement without antibiotics, ABLC reduced the rate of implant infections.^19^,^20^

The use of ABLC in primary arthroplasty is increasing. In Norway, the use of ABLC in primary total hip arthroplasties (THAs) increased from 40% in 1987 to 90% in 1998.^4^ Data from the National Hip Replacement Outcome Project in Britain indicate that 69% of surgeons use ABLC in primary THAs.^21^ Persson *et al*.^22^ showed that antibiotic bone cement reduced the risk of revision surgeries, in a review from the Swedish Joint Registry, and recommended the use of ABLC and systemic antibiotics for prophylaxis. Chiu *et al*.^23^ found a significant decrease in the incidence of infection of total knee replacements when antibiotic cement was used without stratification in a randomized prospective trial. This group also found a decreased incidence of infected total knee replacements in patients with diabetes mellitus when antibiotic cement was used.^18^

In present study, out of 6 infected knees, 3 (2.5% of total 120) were diabetic and 3 (3.75% of total 80) were having rheumatoid arthritis. Out of these six patients, one diabetic patient was treated for an intercondylar fracture of the femur and was having no signs of infection either in the past or at the time of TKA. In contrast, 32 patients had both diabetes and rheumatoid polyarthritis but none of them had infection till the recent follow-up.

Numerous studies have shown that the addition of 1 g of powdered antibiotic to 40 g of PMMA will not significantly affect the fatigue strength of the cement.^24^–^26^ The addition of > 1 g of antibiotic powder per 40 g of PMMA can cause significant weakening of the cement. Lautenschlager *et al*.^27^ demonstrated that adding more than 4.5 g of gentamicin powder per 40-g package of cement caused a loss in the compressive strength below American Society for Testing and Materials standards. This group also showed that the addition of liquid antibiotics caused a substantial loss in cement strength.^28^

Systemic toxicity related to the use of low-dose antibiotic-impregnated cement has not been reported.^29^–^31^ Local toxicity to cells at the interface of the antibiotic cement has not been reported, but few studies have examined changes in the cell function. Pedersen and Lund^32^ examined the effect of the gentamicin-loaded bone cement on mouse calvarial cultures and found that the addition of antibiotic to the cement caused decreased bone turnover presumably through decreased osteoblast and osteoclast function. Isefuku *et al*.^33^ noted that gentamicin levels of > 100 mg/l inhibited osteoblast proliferation in *in vitro* experiments.

Kendall *et al*.^34^ noted survival of bacteria on ABLC *in vitro*. Tunney *et al*.^35^ cultured bacteria from prostheses retrieved at revision surgery (including many patients where no infection was suspected). Wininger and Fass^36^ reported that infection rates at Ohio State University Medical Center decreased with the use of ABLC, but the prevalence of aminoglycoside-resistant bacteria had increased to 20% for S. aureus species, and 60% for S. epidermidis infections. In the present study, we did not grow any organism from the suspected synovial or granulation tissues obtained from the infected knees, hence no opinion regarding the emergence of resistance could be made. Due to increasing evidence of the risk of development of antibiotic resistance, ABLC is being used only for arthroplasty at high risk for the development of infection. The emergence of resistant strains of organisms with the use of ABLC has been minimal, as indicated in the Swedish Registry. The use and type of antibiotic should be recorded if used in the primary TJA. Concerns with the cost-to-benefit ratio are minimal, according to the Swedish data. Projected savings appear to support this use of ABLC, but definitive data have not been shown.

Gentamicin is the most commonly added antibiotic, as it has a broad antibacterial spectrum relevant to joint replacement and elutes from PMMA.^37^ However, not all organisms associated with prosthetic infection will be gentamicin sensitive, and some strains of methicillin-resistant *S. aureus* (MRSA) are also gentamicin resistant. The volume of additional antibiotics needs to be carefully assessed, as too much may significantly reduce the mechanical properties of the cement.^38^,^39^

## CONCLUSION

We support the use of low-dose ABLC as one of the effective means in preventing infection in primary TJAs in both high-risk and nonrisk patients. Extensive data from Norwegian and Swedish hip registries and limited U.S. data indicate that the use of low-dose (less than 2 g of antibiotic per 40 g of cement) ABLC is safe and effective in primary TJAs. Though we do not have any control group but we had no infection in any knee of the patients without risk and we can conclude that ABLC has a preventive role in the nonrisk group also.
